# A novel model to predict positive prostate biopsy based on serum androgen level

**DOI:** 10.1530/ERC-17-0134

**Published:** 2017-10-18

**Authors:** Takeshi Ujike, Motohide Uemura, Atsunari Kawashima, Akira Nagahara, Kazutoshi Fujita, Yasushi Miyagawa, Norio Nonomura

**Affiliations:** 1 Department of UrologyOsaka University Graduate School of Medicine, Suita, Osaka, Japan; 2 Department of Therapeutic Urologic OncologyOsaka University Graduate School of Medicine, Suita, Osaka, Japan

**Keywords:** free testosterone, total testosterone, prostate cancer, prostate biopsy, percent free testosterone

## Abstract

Circulating levels of prostate-specific antigen (PSA) and testosterone are widely used for the detection of prostate cancer prior to prostate biopsy; however, both remain controversial. Effective screening strategies based on quantitative factors could help avoid unnecessary biopsies. Here, we sought to clarify the predictive value of free testosterone (FT) vs total testosterone (TT) in identifying patients likely to have positive biopsies. This study aims to develop a novel model for predicting positive prostate biopsy based on serum androgen levels. This study included 253 Japanese patients who underwent prostate biopsy at our institution. TT and FT, %FT (=FT/TT), age, PSA, prostate volume (PV) and PSA density (PSAD = PSA/PV) were assessed for association with prostate biopsy findings. The following results were obtained. Of 253 patients, 145 (57.3%) had positive biopsies. Compared to the negative biopsy group, the positive biopsy group demonstrated higher age, PSA and PSAD but lower PV, FT and %FT by univariate analysis. Multivariate logistic regression analysis indicated PSA, PSAD and %FT were independent predictors of cancer detection. We developed a predictive model based on PSAD and %FT, for which the area under the curve was significantly greater than that of PSA (0.82 vs 0.66), a well-known predictor. Applying this analysis to the subset of patients with PSA <10 ng/mL yielded similar results. We confirmed the utility of this model in another independent cohort of 88 patients. In conclusion, lower %FT predicted a positive prostate biopsy. We constructed a predictive model based on %FT and PSAD, which are easily obtained prior to biopsy.

## Introduction

Definitive diagnosis of prostate cancer depends on histopathological verification. Prostate biopsy is the only method for definitive diagnosis of prostate cancer. Prostate cancer is usually suspected on the basis of digital rectal examination (DRE) and/or elevated PSA; however, DRE findings have poor sensitivity, limited specificity and high inter-observer variability. According to EAU-ESTRO-SIOG guidelines on prostate cancer (version 2016) (Mottet *et al*. 2016), PSA is a better predictor of prostate cancer than either DRE or transrectal ultrasonography (TRUS).

Although elevated PSA is the most frequent indication for prostate biopsy, mainly because of its high sensitivity, PSA-based diagnostics also have a low specificity, and thus, likely contribute to the increasing frequency of unnecessary biopsies, which should be avoided. Therefore, several clinical factors have been investigated as alternative indicators. Recently, there has been significant interest in developing multiparametric magnetic resonance imaging (mpMRI) to aid in the diagnosis of prostate cancer (Feng *et al*. 2015, Bergdahl *et al*. 2016); however, it remains unclear whether mpMRI could aid in informing decisions regarding prostate biopsy. EAU-ESTRO-SIOG guidelines (Mottet *et al*. 2016) pointed out that mpMRI has limitations similar to those that beset DRE and TRUS, that is, it is subject to inter-observer variability and the heterogeneity in the definitions of positive and negative examinations. In order to reduce harm by avoiding unnecessary biopsies, there is a need for a more effective screening strategy based on quantitative factors obtained before prostate biopsy.

Since the pioneering report of Huggins and coworkers regarding the relationship between prostate cancer progression and androgen in the 1940s (Huggins *et al*. 1942), many studies have assessed the utility of serum androgen measurement (total testosterone (TT), free testosterone (FT)) in prostate cancer screening (Klap *et al*. 2015, Regis *et al*. 2015). While some studies assessed various parameters calculated by absolute androgen concentrations, for example, the ratio of PSA to TT, or of PSA to FT, these associations remain controversial (Porcaro *et al*. 2010, Albisinni *et al*. 2012, Regis *et al*. 2015).

In this study, we focused on not only absolute androgen levels, but also the relative concentration of FT to TT (%FT). In the first step, we investigated whether TT and FT values can be predictive biomarkers for prostate cancer detection upon prostate biopsy in the Japanese population. Next, we established a novel predictive model, using quantitative factors that can be easily obtained prior to prostate biopsy.

## Materials and methods

### Patients

This study included 253 patients who underwent initial prostate biopsy in Osaka University Hospital from July 2014 to September 2016. The indication for prostate biopsy was suspicion of prostate cancer on the basis of serum PSA elevation and/or DRE findings. None of these patients underwent testosterone therapy. All patients underwent transrectal ultrasound-guided systemic 12-core prostate needle biopsy. Targeted biopsies were conducted for suspicious lesions. To confirm our results in another independent cohort, we recruited 88 patients who underwent prostate biopsy from October 2016 to June 2017 in our institution. This study was approved by the Osaka University Hospital Institutional Review Board.

### Clinical data collected

The following clinical data were collected retrospectively from the medical records at Osaka University Hospital: histopathological findings of prostate biopsy, age, PSA, PV, PSAD, values of serum TT and FT. %FT was obtained by dividing the two concentrations (FT/TT), after converting free testosterone values to ng/mL. PV was measured by TRUS.

### Blood samples

Blood samples were obtained between 08:00 h and 10:00 h to assess TT and FT levels according to the Endocrine Society’s guidelines (Rosner *et al*. 2007). Serum TT was measured by chemiluminescent immunoassay (using Lumipulse Presto Testosterone, Fujirebio Inc., Japan). Serum FT was measured by radioimmunoassay (using Free Testosterone RIA kit, Sceti Medical Labo K.K., Japan).

### Statistical methods

Results were expressed as the median (range) for continuous variables. Univariate analysis was performed by the Mann–Whitney *U* test. Univariate and multivariate logistic regression analysis were performed to determine the correlation between prostate cancer detection and clinical factors (age, PSA, PV, PSAD, TT, FT, %FT). As the variable PSAD is made up of PSA and PV, PV was removed from the models. The predicted probability of a positive biopsy result was estimated as *P* = 1/(1 + *e*^−^*^x^*). Logistic regression yields a score (*X*), where *X* is *β*_0_ + *β*_1_*X*_1 _+ *β*_2_*X*_2 _+ *β*_3_*X*_3_…, which is a linear combination of the predictors (*X*_1_, *X*_2_, *X*_3_…) in the model. The model coefficients (*β*_0_, *β*_1_, *β*_2_) were chosen to optimize the ability to predict a positive biopsy result. A nomogram predicting the probability of prostate cancer was constructed based on this formula. The new diagnostic model obtained was evaluated for diagnostic ability using the receiver-operator characteristics (ROC) curve analysis. To determine significant differences in the area under the ROC curve (AUC) compared to other existing factors, the chi-square test was used. Statistical significance was considered as *P* < 0.05. All data analyses were performed with JMP, ver.10 (SAS Institute Inc., Cary, NC, USA).

## Results

### Analysis in the entire cohort

Patient characteristics are summarized in Table 1. Among 253 patients, 145 patients (57.3%) had a positive biopsy, and 108 patients (42.7%) had a negative biopsy. In univariate analysis, age, PSA and PSAD were significantly higher in the positive biopsy group compared with the negative biopsy group. However, PV, FT and %FT were significantly lower in the positive biopsy group compared with the negative biopsy group. TT was not significantly different between two groups. Multivariate logistic regression analysis revealed that PSA, PSAD and %FT were independent predictors for incidence of prostate cancer upon prostate biopsy (*P* < 0.05), whereas age and FT were not significant (Table 2).

**Table 1:** Patient characteristics of all cases.

**Variables**	**Negative biopsy**	**Positive biopsy**	***P* value**
Number	108	145	
Age (years)	67.5 (35–79)	71 (51–84)	0.0001
PSA (ng/mL)	6.90 (1.06–27.29)	9.67 (2.77–3534.41)	<0.0001
PV (mL)	33.3 (10.5–119.7)	24.7 (10–100)	<0.0001
PSAD (ng/mL/cm^3^)	0.21 (0.05–0.53)	0.42 (0.05–39.5)	<0.0001
TT (ng/mL)	2.98 (1.02–7.76)	3.32 (1.63–9.42)	0.199
FT (pg/mL)	7.4 (2.2–27.5)	6.5 (0.8–17.1)	0.0004
%FT (%)	0.245 (0.081–1.26)	0.196 (0.019–0.481)	<0.0001

Median (range). *P* value was calculated by Mann–Whitney *U* test.

FT, free testosterone; %FT, percent free testosterone; PSA, prostate-specific antigen; PSAD, PSA density; PV, prostate volume; TT, total testosterone.

**Table 2:** Logistic analysis of variables associated with cancer detection in all cases.

**Variables**	**Univariate**			**Multivariate**		
	OR	95% CI	*P* value	OR	95% CI	*P* value
Age	1.08	1.04–1.13	<0.0001	1.02	0.97–1.08	0.343
PSA	1.09	1.05–1.14	<0.0001	0.93	0.90–1.00	0.048
PV	0.97	0.95–0.98	<0.0001			
PSAD 0.1 increase	1.87	1.53–2.36	<0.0001	2.08	1.60–2.76	<0.0001
TT	1.12	0.92–1.38	0.274			
FT	0.84	0.76–0.92	0.0001	0.99	0.86–1.14	0.917
%FT 0.01 increase	0.92	0.89–0.96	<0.0001	0.93	0.89–0.98	0.0042

When treating PSAD and %FT as continuous predictors, the odds ratio have been computed for a 0.1 increase in PSAD levels and a 0.01% increase in %FT levels. *P* value was calculated by likelihood ratio test.

CI, confidence interval; FT, free testosterone; %FT, percent free testosterone; OR, odds ratio; PSA, prostate-specific antigen; PSAD, PSA density; PV, prostate volume; TT, total testosterone.

**Figure 1:**
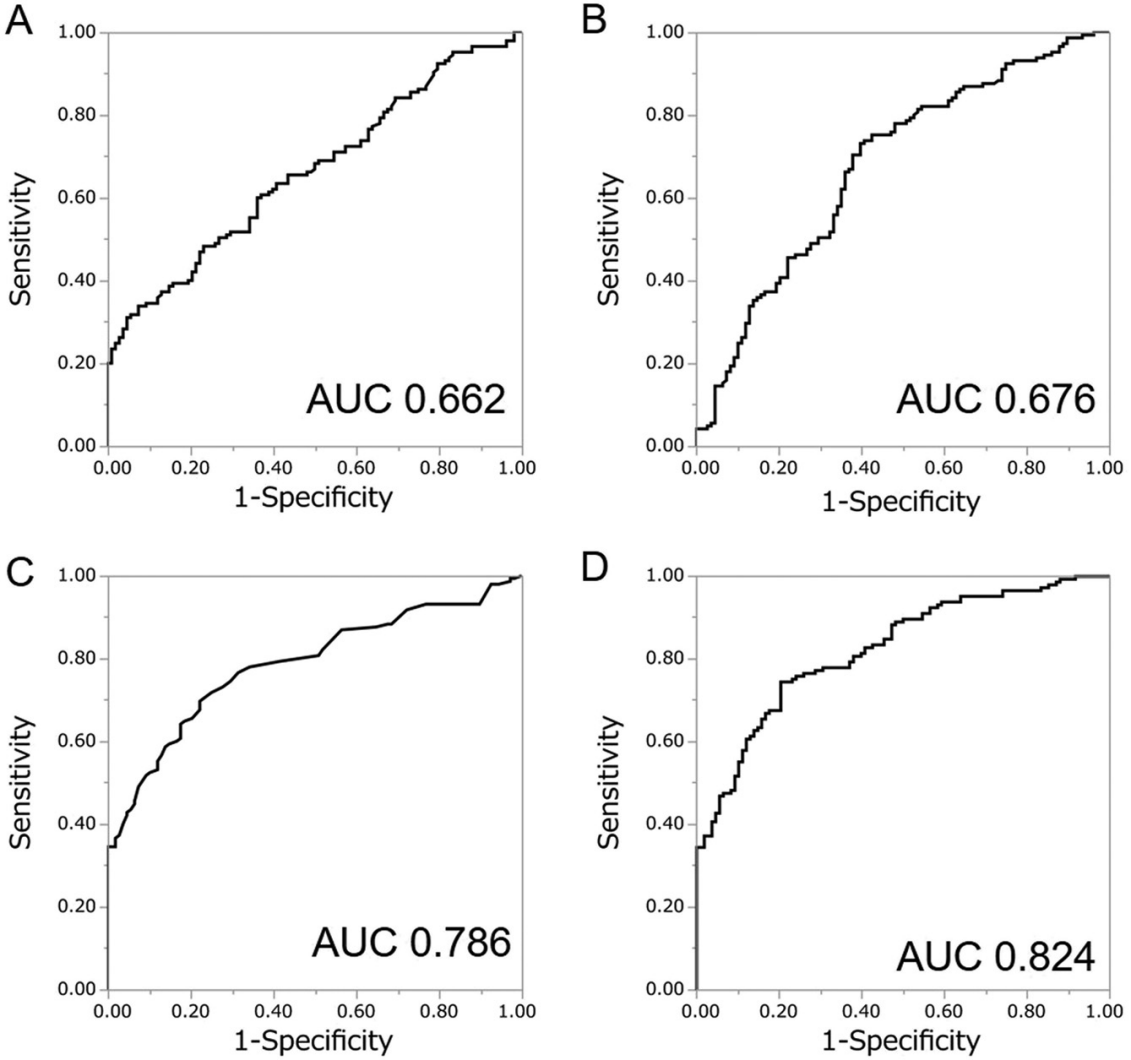
Receiver-operator characteristics (ROC) curves of the predicted probability of prostate cancer detection for all 253 patients by PSA (A), %FT (B), PSAD (C) and PSAD-%FT model (D).

**Figure 2:**
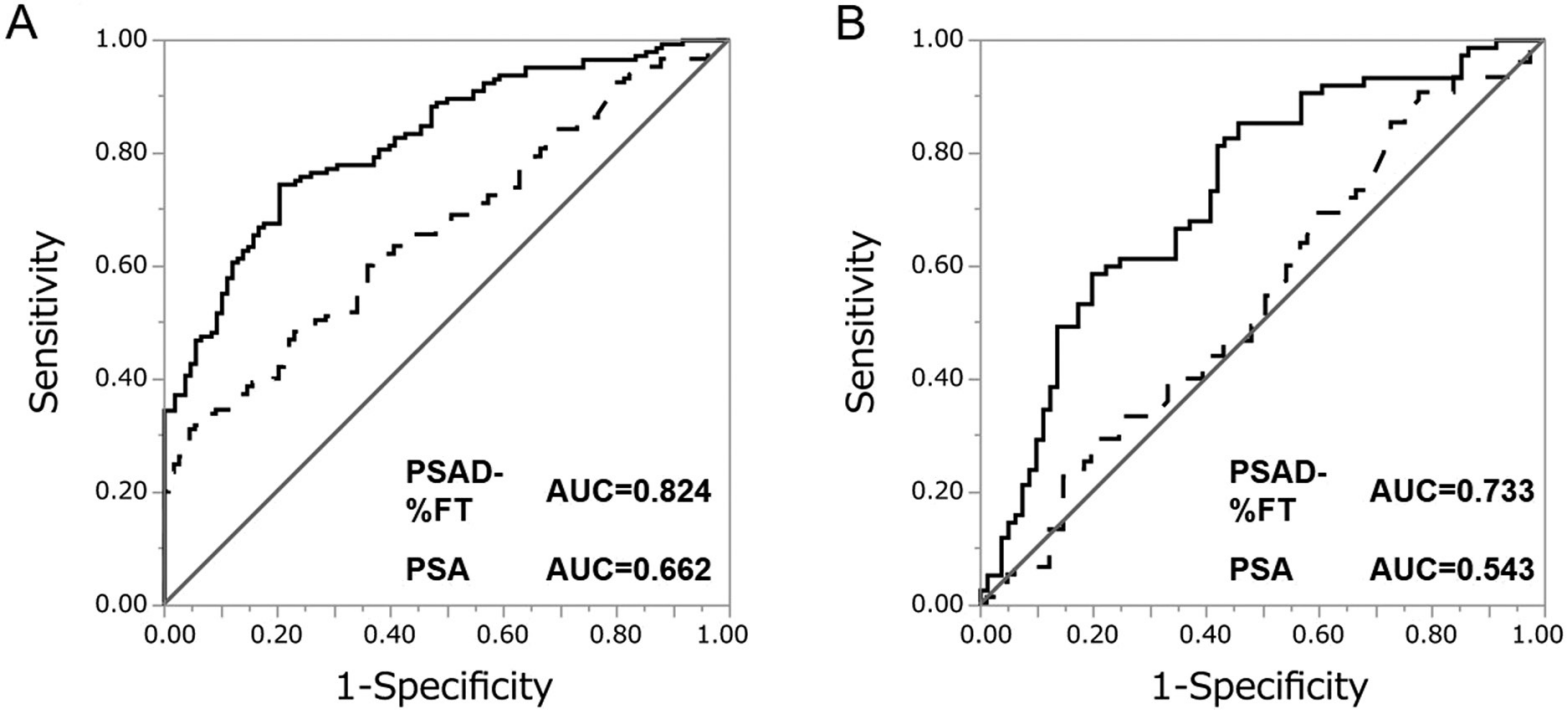
Receiver-operator characteristics (ROC) curves of the predicted probability of prostate cancer detection by the PSAD-%FT model (solid curves) and PSA (dotted curves). (A) ROC curves for all 253 patients. (B) ROC curves for 156 patients with PSA levels under 10 ng/mL.

Subsequently, from the result of multivariate logistic regression analysis using PSAD and %FT, we created a predictive model for the probability of detecting prostate cancer upon biopsy as represented by the following formula: *P* = 1/(1 + *e*^−^*^x^*) *X* = 0.198 − 7.96 × %FT + 5.85 × PSAD. Using this predictive model (PSAD-%FT model), the AUC for the probability of detecting prostate cancer in all patients was 0.824, while the AUC for PSA, %FT and PSAD were 0.662, 0.676 and 0.786, respectively (Fig. 1). The AUC for PSAD-%FT model was statistically greater than PSA (*P* < 0.0001) (Fig. 2A) and PSAD (*P* = 0.018). The sensitivity and specificity of PSAD-%FT model was 74.5% and 79.7%, respectively.

### Analysis in the subgroup of patients with a PSA level under 10 ng/mL

We then repeated this analysis among the subset of patients with PSA <10 ng/mL. Among these 156 patients, 75 patients (45.5%) had a positive biopsy and 81 patients (54.5%) had a negative biopsy. These patient characteristics are summarized in Table 3. In univariate analysis, PSAD was significantly higher in the positive biopsy group compared with the negative biopsy group, while PV, FT and %FT were significantly lower in the positive biopsy group compared with the negative biopsy group. Multivariate logistic regression analysis revealed that PSAD and %FT were independent predictors of outcome of prostate biopsy (*P* < 0.05) (Table 4).

**Table 3:** Patient characteristics of 156 patients with PSA levels under 10 ng/mL.

**Variables**	**Negative biopsy**	**Positive biopsy**	***P* value**
Number	81	75	
Age (years)	67 (35–79)	71 (52–80)	0.014
PSA (ng/mL)	5.75 (1.06–9.93)	5.74 (2.77–9.74)	0.358
PV (mL)	31 (10.5–90)	24 (10–77)	0.0004
PSAD (ng/mL/cm^3^)	0.19 (0.05–0.52)	0.27 (0.05–0.65)	0.0002
TT (ng/mL)	3.02 (1.35–7.76)	3.32 (1.86–6.81)	0.378
FT (pg/mL)	7.8 (2.2–27.5)	6.6 (0.8–17.1)	0.003
%FT (%)	0.249 (0.081–1.27)	0.195 (0.019–0.353)	<0.0001

Median (range). *P* value was calculated by Mann–Whitney *U* test.

FT, free testosterone; %FT, percent free testosterone; PSA, prostate-specific antigen; PSAD, PSA density; PV, prostate volume; TT, total testosterone.

**Table 4:** Logistic analysis of variables associated with cancer detection in 156 patients with PSA levels under 10 ng/mL.

**Variables**	**Univariate**			**Multivariate**		
	OR	95% CI	*P* value	OR	95% CI	*P* value
Age	1.06	1.01–1.12	0.014	1.03	0.98–1.10	0.236
PSA	1.07	0.91–1.26	0.421			
PV	0.96	0.94–0.97	0.002			
PSAD 0.1 increase	1.72	1.29–2.36	0.0002	1.55	1.15–2.15	0.004
TT	1.08	0.83–1.42	0.556			
FT	0.84	0.73–0.94	0.002	0.99	0.85–1.16	0.930
%FT 0.01 increase	0.91	0.87–0.96	<0.0001	0.93	0.88–0.98	0.009

When treating PSAD and %FT as continuous predictors, the odds ratio have been computed for a 0.1 increase in PSAD levels and a 0.01% increase in %FT levels. *P* value was calculated by likelihood ratio test.

CI, confidence interval; FT, free testosterone; %FT, percent free testosterone; OR, odds ratio; PSA, prostate-specific antigen; PSAD, PSA density; PV, prostate volume; TT, total testosterone.

**Figure 3:**
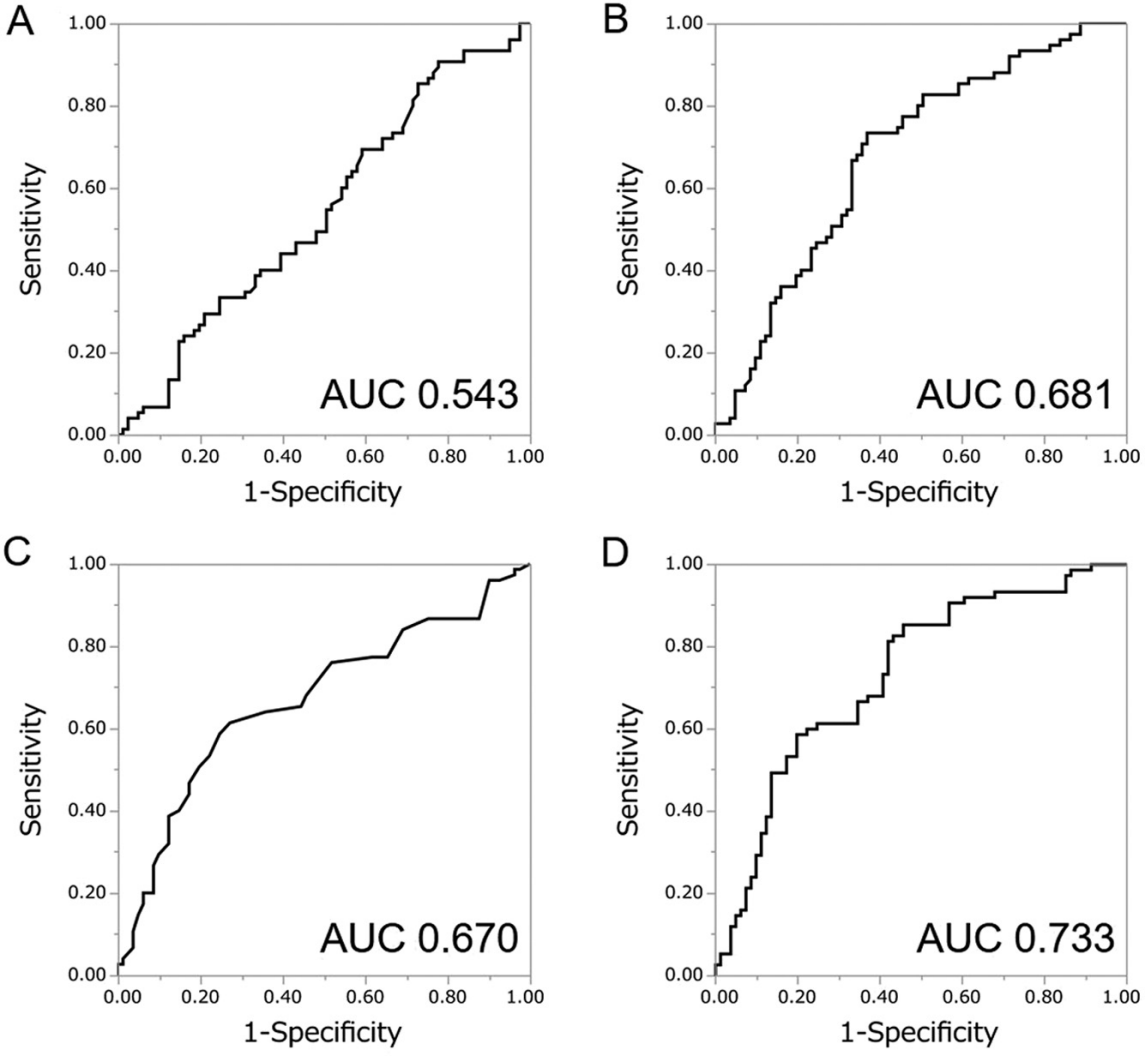
Receiver-operator characteristics (ROC) curves of the predicted probability of prostate cancer detection for 156 patients with PSA levels under 10 ng/mL by PSA (A), %FT (B), PSAD (C) and PSAD-%FT model (D).

Similar to the previous analyses, we created a predictive model by combining these two predictors (PSAD and %FT) for this subgroup analysis based on the result of multivariate logistic regression analysis using PSAD and %FT. The following model for predicting the probability of detecting prostate cancer by prostate biopsy was obtained: *P* = 1/(1 + *e*^−^*^x^*) *X* = 0.650 − 8.13 × %FT + 4.61 × PSAD. Using this predictive model (PSAD-%FT model), the AUC for the probability of detecting prostate cancer was 0.733, while the AUC for PSA, %FT and PSAD were 0.543, 0.681 and 0.670, respectively (Fig. 3). The AUC for PSAD-%FT model was greater than that for PSA (*P* = 0.0044) and PSAD (*P* = 0.06) (Fig. 2B). The sensitivity and specificity of this optimal model were 85.3% and 54.3%, respectively.

### Validation in testing cohort

Next, in order to confirm the utility of this model (PSAD-%FT model), we applied it to 88 patients in an independent cohort.

**Figure 4:**
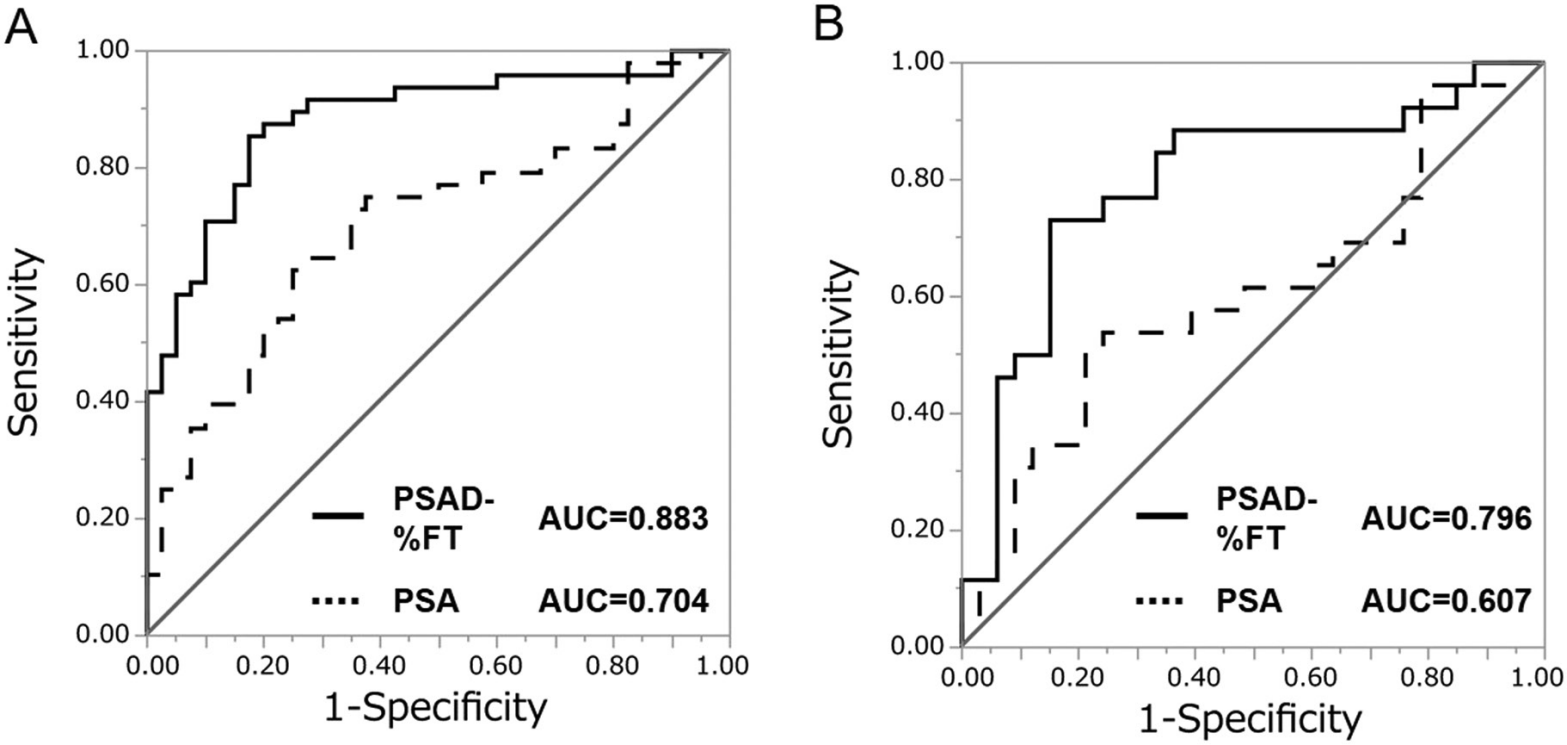
Receiver-operator characteristic (ROC) curves of the predicted probability of prostate cancer detection by the PSAD-%FT model (solid curves) and PSA (dotted curves) in a testing cohort. (A) ROC curves for all 88 patients. (B) ROC curves for 59 patients with PSA levels under 10 ng/mL.

Patient characteristics are summarized in Table 5. Among 88 patients, 48 patients (54.5%) had a positive biopsy, and 40 patients (45.5%) had a negative biopsy. In this cohort, ROC curve analysis was performed by applying the following predictive formula: *P* = 1/(1 + *e*^−^*^x^*) *X* = 0.198 − 7.96 × %FT + 5.85 × PSAD. Using this predictive model (PSAD-%FT model), the AUC for the probability of detecting prostate cancer was 0.883. The AUC for PSAD-%FT model was greater than that for PSA (AUC = 0.704, *P* = 0.0003) (Fig. 4A) and PSAD (AUC = 0.854, *P* = 0.286).

**Table 5:** Patient characteristics in testing cohort.

**Variables**	**Negative biopsy**	**Positive biopsy**	***P* value**
Number	40	48	
Age (years)	69 (50–81)	72 (57–84)	0.138
PSA (ng/mL)	6.38 (3.55–29.41)	9.70 (4.20–408)	0.0011
PV (mL)	35.6 (17.3–139.8)	26.0 (12.2–72)	0.0002
PSAD (ng/mL/cm^3^)	0.18 (0.06–0.41)	0.37 (0.09–5.67)	<0.0001
TT (ng/mL)	3.52 (1.65–7.31)	3.56 (1.82–7.36)	0.821
FT (pg/mL)	7.8 (3.1–16.7)	7.1 (3.4–15.2)	0.419
%FT (%)	0.207 (0.107–0.315)	0.193 (0.103–0.298)	0.0588

Median (range). *P* value was calculated by Mann–Whitney *U* test.

FT, free testosterone; %FT, percent free testosterone; PSA, prostate-specific antigen; PSAD, PSA density; PV, prostate volume; TT, total testosterone.

In our testing cohort, we also added an analysis among the subset of patients with PSA <10 ng/mL. Patient characteristics are summarized in Table 6. Among 59 patients, 26 patients (44.1%) had a positive biopsy, and 33 patients (55.9%) had a negative biopsy. In this cohort, ROC curve analysis was performed by applying the following predictive formula: *P* = 1/(1 + *e*^−^*^x^*) *X* = 0.650 − 8.13 × %FT + 4.61 × PSAD.

**Table 6:** Patient characteristics in testing cohort with PSA levels under 10 ng/mL.

**Variables**	**Negative biopsy**	**Positive biopsy**	***P* value**
Number	33	26	
Age (years)	71 (50–81)	72 (60–79)	0.515
PSA (ng/mL)	5.92 (3.55–9.90)	6.99 (4.20–9.91)	0.160
PV (mL)	33.0 (17.3–84.8)	25.7 (12.9–52.2)	0.014
PSAD (ng/mL/cm^3^)	0.17 (0.06–0.41)	0.25 (0.09–0.67)	0.0006
TT (ng/mL)	3.55 (1.65–7.31)	3.64 (2.03–7.36)	0.541
FT (pg/mL)	7.9 (3.1–16.7)	6.9 (3.8–12.8)	0.725
%FT (%)	0.207 (0.107–0.315)	0.186 (0.126–0.298)	0.0211

Median (range). *P* value was calculated by Mann–Whitney *U* test.

FT, free testosterone; %FT, percent free testosterone; PSA, prostate-specific antigen; PSAD, PSA density; PV, prostate volume; TT, total testosterone.

Using this predictive model (PSAD-%FT model), the AUC for the probability of detecting prostate cancer was 0.796. The AUC for PSAD-%FT model was greater than that for PSA (AUC = 0.607, *P* = 0.0114) (Fig. 4B) and PSAD (AUC = 0.762, *P* = 0.566).

In summary, analysis of the validation cohort resulted in substantially the same findings as from the original cohort: in addition to higher PSAD, which is a well-known predictor, %FT levels predict a positive biopsy for prostate cancer.

## Discussion

After Huggins and coworkers reported that metastatic prostate cancer growth was suppressed by eliminating androgens via castration, it was believed for a long time that higher TT contributed to prostate cancer and caused rapid cancer growth (Huggins *et al*. 1942).

However, recent studies have demonstrated no relationship between TT levels and prostate cancer risk (Mohr *et al*. 2001, Yano *et al*. 2007, Morote *et al*. 2009, Botelho *et al*. 2012). Yano and coworkers investigated the relationship between TT levels and positive biopsy among 420 patients and concluded that there was no significant difference in pretreatment TT among the positive and negative biopsy groups (Yano *et al*. 2007). Rather paradoxically, some studies reported a significant correlation between low levels of TT with increased prostate cancer risk or grade (Shin *et al*. 2010, Garcia-Cruz *et al*. 2012, Tu *et al*. 2017).

Shin and coworkers investigated prostate cancer risk on prostate biopsy according to TT level (Shin *et al*. 2010). In their study, 568 patients were enrolled and divided into two groups according to median TT level (385 ng/mL). They concluded that patients with lower levels of TT had a higher risk of prostate cancer in the Korean population.

Recently, active surveillance has become one of the most important treatment options for low-risk prostate cancer, but no firm criteria have been established to guide this decision. Some studies identified clinical factors associated with tumor upgrading in low-risk prostate cancer (Porcaro *et al*. 2016, 2017, Ferro *et al*. 2017). Ferro and coworkers and Porcaro and coworkers reported the usefulness of TT measurement in informing decisions regarding active surveillance, but did not come to definitive conclusions (Ferro *et al*. 2017; Porcaro *et al*. 2017).

At the present time, correlation between serum TT level and prostate cancer risk is controversial. Clap and coworkers reviewed 45 articles that discussed TT and the subsequent risk of prostate cancer, concluding that the literature reports contradictory results: Of the 45 articles, 18 suggested low TT increases the risk of prostate cancer, 17 suggested high TT increases the risk and 10 showed no relationship (Clap *et al*. 2015). Much of this controversy appears to be based on conflicting study designs, definitions and methodologies. The review also pointed out that these conflicting results may stem from insufficient knowledge about the underlying physiology of prostate cancer and that the effective concentration of TT in prostate tissue must be considered.

In terms of endocrinology, much of the circulating TT in the blood is bound to protein and not available to cells. To investigate the relationship between androgens and prostate cancer risk, we focused on FT, which is bioavailable and a measurable component in TT. TT concentration refers to both bioavailable and non-bioavailable testosterone in the circulation. The term ‘bioavailable testosterone’ represents the sum of FT plus testosterone bound loosely to albumin. FT represents only 0.5–3% of TT, but FT is considered the more biologically active form. In Japan, FT values are used in the diagnosis late-onset hypogonadism (LOH) (Namiki *et al*. 2008).

In young men, FT represents about 2–3% of TT (De Ronde *et al*. 2006). As men age, although TT declines (0.4%/year), FT declines to a greater extent (1.2%/year) (Swerdloff *et al*. 2004), resulting in a lower %FT in older men. In this study, the median age is 69 years (range 35–84) and the median %FT is 0.21% (range 0.019–1.27%).

The relationship of FT to prostate cancer treatment and outcomes remains largely unexplored, with only a few studies investigating a possible connection (Hoffman *et al*. 2000, Pierorazio *et al*. 2010, Garcia-Cruz *et al*. 2013, Leon *et al*. 2015). Hoffman and coworkers retrospectively reviewed 117 patients diagnosed with prostate cancer (Hoffman *et al.* 2000). They reported that all men with a Gleason score of 8 or greater on their prostate biopsy had low free testosterone. They concluded that this finding suggested low serum free testosterone is a marker of more aggressive disease. Leon and coworkers prospectively assessed whether preoperative circulating testosterone levels, obesity and metabolic syndrome were correlated with aggressive pathological features after robotic prostatectomy (Leon *et al.* 2015). As a result of examining 354 patients undergoing robot-assisted prostatectomy, they concluded that low FT levels were linked with high-grade prostate cancer.

In total, the relationship between prostate cancer risk and absolute androgen concentration remain controversial. In the present study, we focused not only on TT and FT, but also on the FT/TT ratio (i.e., %FT). A survey of the most current literature includes only one study that examines the utility of %FT as a predictor for high-grade prostate cancer in men undergoing prostate biopsy (Albisinni *et al*. 2012). They collected data on 812 white Italian men who underwent prostate biopsy and analyzed the association between prostate biopsy and serum androgen concentrations. They conclude that a greater %FT level was associated with an increased risk of high-grade prostate cancer (Gleason score ≥7), but not low-grade prostate cancer (Gleason score ≤6).

Our data show that low %FT could be a predictor for positive prostate biopsy. These findings suggest that although absolute androgen levels do not modify prostate cancer risk, the free-to-total testosterone ratio could be predictive of positive prostate biopsy. Future studies are needed to address whether %FT adds meaning to risk stratification for prostate cancer risk and to determine the underlying biologic basis for these results.

Prostate biopsy is the only method to confirm the diagnosis of prostate cancer. Before a prostate biopsy is carried out, it is difficult to discriminate clearly between prostate cancer and benign prostate hyperplasia by PSA values alone. Therefore, several clinical factors and tumor markers have been studied. Some studies reported the utility of Prostate Health Index (Phi) as calculated by ProPSA (Liang *et al*. 2011, Lazzeri *et al*. 2012, Bruzzese *et al*. 2014), prostate cancer gene-3 (PCA3) (Ploussard *et al*. 2010, Elshafei *et al*. 2015), gene polymorphisms (Singh *et al*. 2005, Akamatsu *et al*. 2012) and multiparametric MRI (Feng *et al*. 2015, Bergdahl *et al*. 2016). However, we hoped to develop a quantitative and inexpensive marker in the clinic. We developed a novel predictive model for positive prostate biopsy based on PSAD and %FT, as derived from readily available PSA, PV, TT and FT values. These values can be measured easily and inexpensively.

In this study, some limitations should be taken into consideration. Firstly, this study consisted of only Japanese patients, and these results might not be applied to other races. Secondly, the number of patients in this study was relatively small. However, we strongly believed that these interesting results can help clinicians in deciding whether prostate biopsy should be carried out, especially for patients with no other evidence than elevated PSA. The true correlation between low levels of %FT and prostate cancer risk needs validation in large cohort prospective studies.

## Conclusion

Percent-FT (FT/TT ratio), which can be measured easily and inexpensively, was found to be a good predictor of prostate cancer upon prostate biopsy, whereas serum TT and FT values were not. Low %FT level is an independent risk factor for prostate cancer detection. We were able to construct a novel predictive model based on %FT and PSAD, which are quantitative factors obtained before prostate biopsy. This model can assist clinicians in deciding whether prostate biopsy is advisable.

## Declaration of interest

The authors declare that there is no conflict of interest that could be perceived as prejudicing the impartiality of the research reported.

## Funding

This research did not receive any specific grant from any funding agency in the public, commercial or not-for-profit sector.
